# Surface-Activated Pencil Graphite Electrode for Dopamine Sensor Applications: A Critical Review

**DOI:** 10.3390/bios13030353

**Published:** 2023-03-06

**Authors:** Sakthivel Srinivas, Annamalai Senthil Kumar

**Affiliations:** 1 Department of Chemistry, School of Advanced Sciences, Vellore Institute of Technology, Vellore 632 014, India; 2 Nano and Bioelectrochemistry Research Laboratory, Carbon Dioxide Research and Green Technology Centre, Vellore Institute of Technology, Vellore 632 014, India

**Keywords:** pencil graphite electrode, surface activation procedures, pre-anodization, polymer-modified electrode, metal nanoparticle-modified electrode, dopamine sensor

## Abstract

Pencil graphite electrode (PGE) is an alternative, commercially available, ready-to-use, screen-printed electrode for a wide range of electroanalytical applications. Due to the complex-matrix composition and unpredictable electro-inactive nature of PGE in its native form, a surface pre-treatment/activation procedure is highly preferred for using it as an electroactive working electrode for electroanalytical applications. In this article, we review various surface pre-treatment and modification procedures adopted in the literature with respect to the sensitive and selective detection of dopamine as a model system. Specific generation of the carbon–oxygen functional group, along with partial surface exfoliation of PGE, has been referred to as a key step for the activation. Based on the Scopus^®^ index, the literature collection was searched with the keywords “pencil and dopamine”. The obtained data were segregated into three main headings as: (i) electrochemically pre-treated PGE; (ii) polymer-modified PGEs; and (iii) metal and metal nanocomposite-modified PGE. This critical review covers various surface activation procedures adopted for the activation for PGE suitable for dopamine electroanalytical application.

## 1. Introduction

The use of a low-cost disposable type of electrode in electrochemistry and electrochemical applications holds continued research interest. In this connection, screen-printed electrode (SPE) [1], pencil [2], pencil-drawn paper [3] and systems based on cellulose sheet [4], plastic [5], thread [6] etc. have been widely referred. Amongst all, the pencil graphite-based electrodes, designated as pencil graphite electrode (PGE), have added advantages over the other systems in terms of easy, ready-to-use, commercial availability and reusable quality [7]. PGE has been utilized as a reliable electrode for various electrochemical sensing applications, noticeably to organic pollutants [8], heavy metal detection [9], pharmaceutical drug analyses [10], etc. Different grade pencils of variable dimensions, such as 0.5 mm, 0.7 mm, 1 mm, 2 mm, etc., were available commercially. In general, an H-grade PGE consists of nearly 65% graphite, 30% clay and 5% wax and other electro-inactive substances [11]. Based on the content, the graphite pencils are labelled with letters H (hardness) and B (blackness) and numbered with the degree of hardness/blackness from 9H (the softest) to 8B (the hardest). Graphite-based materials derived from PGE by different methods, such as chemical etching [2], exfoliation [12] and over-oxidations [13], exhibit a wide range of electrochemical properties, such as electrochemical supercapacitor, batteries and sensing applications. Meanwhile, in the literature, there were few review articles covering a general overview of the pencil system in electrochemical and electroanalytical chemistry [2,7,14]. In this work, owing to the biomedical importance, we for the first time reviewed the pencil-based electrodes used for the dopamine electroanalysis covering an in-site view of PGE activation and chemical modification procedures.

Dopamine (DA), a catecholamine-based reversible redox system, is an important neurotransmitter present in the brain and is involved in neural functions and transducing nerve signaling [15]. Abnormal DA secretion affects the hormonal and emotional stability of humans [16]. However, DA deficit in the brain causes Parkinson’s disease and schizophrenia [17]. In general, spectroscopic techniques, such as gas-chromatography (GC-MS) [18], high-pressure liquid chromatography (HPLC) [19] and colorimetric (UV-Vis) [20] methods, are referred for DA analysis. The above higher-ended spectroscopic methods demand time-consuming sample preparation methods and skilled human resources. Electroanalytical methods are a great alternative choice, having advantages such as easy sample preparation and cost-effective and instant results. In the past two decades, the development of electrochemical voltammetric techniques and the fabrication of low-cost modified electrodes for sensitive and selective DA estimation in body fluids has gained attention; ascorbic acid (AA) and uric acid (UA) interference-free detection of DA has been an especially challenging task [21,22,23,24,25,26,27]. Various chemically modified electrodes have been constructed based on organic redox mediators [28], polymers-modified electrodes [29,30], metal nanocomposites [31,32,33] and nanoparticle-modified electrodes [34]. This work covers detailed literature information about the nature of PGE and its activation techniques adopted in the literature for the DA electroanalysis.

**Scheme 1:**
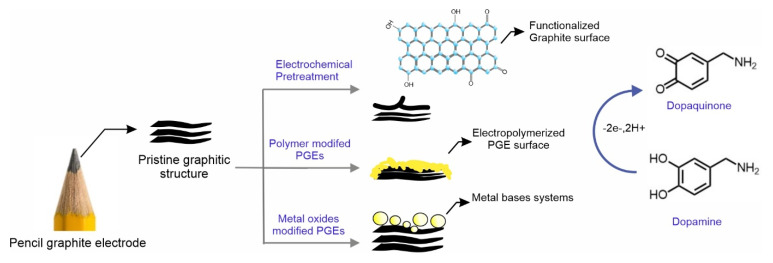
Schematic representation of the work illustrating the skeleton of this review article relating to dopamine electrochemical oxidation and sensing.

It has been reported that the composition of graphite and clay added to the pencil influences electrochemical and structural (mechanical strength) properties [35]. For instance, a B-grade pencil electrode was used for the detection of boron in the presence of tiron [36], 6B was optimized for the sensitive and selective detection of phenols [11] and HB pencil was reported for polyphenolic acids electroanalysis [37,38]. Indeed, in general, untreated PGE is not suitable for electrochemical and electroanalytical activities. It needs surface pretreatment for the activation (PGE*, * = activated surface), which may result in an improved electroconductivity, electroactive surface area and adsorptive nature. For the activation of PGE, anodization [39], modification of organic polymers [40], metal oxides [41], nanoparticles [42] and nanomaterial [43] have been referred. The database used in the search of the research articles is sourced from Scopus^®^ with keywords in the title as “pencil, dopamine”. Published articles were categorized into three major subdivisions as: (i) electrochemically pre-treated PGE, (ii) polymer-modified PGEs and (ii) metal oxides and nanocomposites-modified PGE as represented in Scheme 1. This review article highlights the activation techniques adopted and details of the DA electroanalysis. In the end, the overall conclusion and future prospects of the PGE toward DA were covered. 

## 2. Activated Pencil Graphite Modified Electrodes for Electrochemical DA Sensing Applications

### 2.1. Electrochemically Pre-Treated PGE

**Scheme 2:**
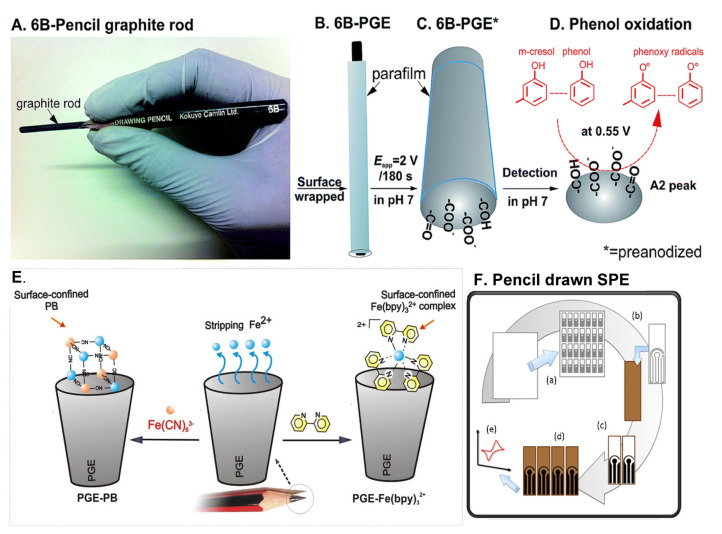
(**A**,**B**) Cartoon created to represent the preparation of PGE for electrochemical analysis. (**C**,**D**) Schematic representation for surface functionalization and catalytic effect towards substituted phenols. (**E**) Mechanism proposed for iron–bypyridyl complex formation on PGE. (**F**) Step-by-step preparation of low–cost screen-printed electrode using PGE. Reprinted with permission from RSC and Elsevier publishers [11,64,65].

**Scheme 3:**
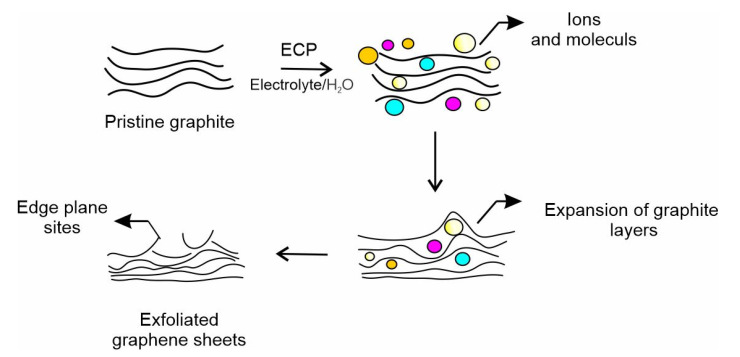
Proposed cartoon illustrating the mechanism of edge plane site formation on graphite surface during electrochemical treatment. ECP = Electrochemical procedure.

**Figure 1:**
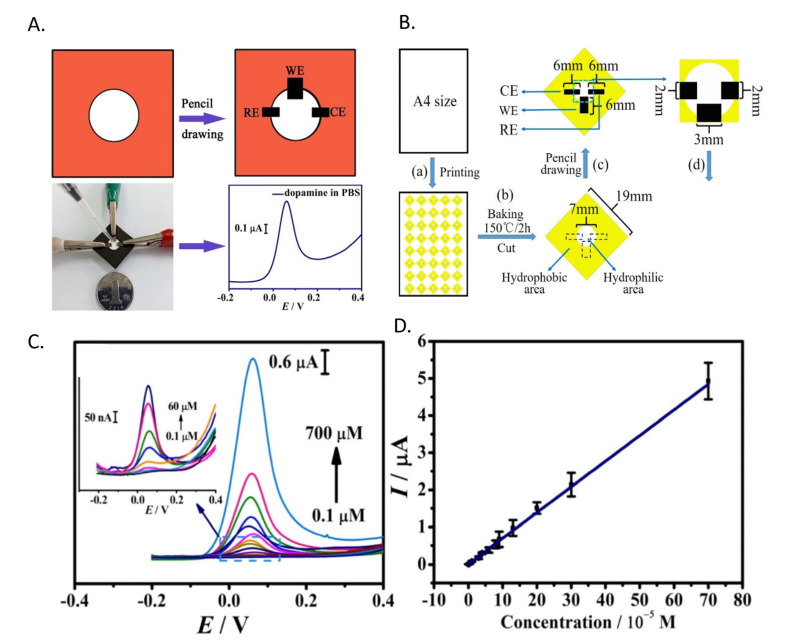
(**A**) Diagrammatic representation of fully drawn paper electrode and its DPV response. (**B**) Measurement sketch for the preparation of electrode. (**C**) DPV response of PGE/PDA modified electrode in presence of DA (0.1–700 µM) in PBS 7 solution and (**D**) its respective calibration plot *i*_p_ vs. [DA]/M. Reprinted with permission of Elsevier publisher [3].

Electrochemical surface pretreatment plays a vital role in improvising the electroanalytical performance and delinking the masked binding agents that could hinder the electrochemical reaction. However, different surface treatments express different functionality towards its selectivity, sensitivity and conductivity. There were few surface treatments/activation procedures reported for the generation of various carbon–oxygen surface functional groups on the graphitic structures relating to dopamine (DA) sensing applications (Table 1; Scheme 2A–F). Scheme 3 illustrates the general electrochemical route for the surface activation of graphite via new edge plane site formation. The edge plan contains a variety of carbon–oxygen/sp^3^ functional groups (sp^3^ carbons) suitable to form weak adsorption with analytes via multiple hydrogen bondings and to facilitate the electron-transfer reaction. The electrochemical response of DA varies depending on the nature of the graphitic surface, basal plane and edge plane graphitic sites. Heterogenous electron transfer kinetics has been used as a tool to reveal the basal and edge plane sites of the graphitic surface in the literature. Higher kinetics and lesser adsorption behavior of DA have been noticed on the edge plane sites over the basal plane sites, whereas the absence of any electrocatalysis response was observed on basal plane graphitic sites (likely due to electroinactive polymer formation) [41,44,45]. Hence, DA has been used as a model electrochemical probe to understand the surface feature of graphitic materials [46]. In 2008, Valencia et al. first reported the simultaneous determination of DA, AA and serotonin (SE) using a PGE* [47]. A homemade sensor was prepared manually using a micropipette and epoxy resin, where the surface activation of the PGE was carried out by performing 20 continuous cyclic voltammetric measurements in a potential window of −0.6 to +0.9 V vs SCE in a N_2_ purged 0.1 M phosphate buffer solution (PBS) at *v* = 100 mVs^−1^. Unfortunately, no physicochemical characterization of the PGE* was demonstrated. Electrochemical oxidative detection of DA was measured using differential pulse voltammetry (DPV) with a marked peak-to-peak separation potential between DA and AA (~70 mV). The electrode showed a good sensitivity 4.03 µA mM^−1^ but with a short linear range of 0.1–0.7 mM for DA. The limited linear range could be the reason that the real sample analysis was not demonstrated in this work. A separation-free and simultaneous monitoring of DA with other biochemicals, such as AA and UA for real time applications, are of great interest. Indeed, the overlapping of the oxidation potentials and the difficulty in resolving the voltammetric peaks are challenging. In this connection, adsorptive stripping voltammetry technique (AdSV), wherein the analytes were selectively adsorbed on the working electrode at a pre-set operating potential in the first step, followed by stripping of the adsorbed species at a suitable scanning potential as a second step, was adopted. In 2009, Ozcan and Sahin reported an AdSV-based detection of DA in the presence of AA and UA in pH 8.5 PBS [48]. Prior to the analysis, the PGE (0.5 mm) surface was activated by potential sweeping it at a window of −0.3 to +2 V vs SCE in 0.1 M H_3_PO_4_. In their experiment, the step 1 preconcentration procedure was carried out by exposing the analytes to an open circuit condition for about 180 s in pH 4 PBS solution followed by stripping analysis under hydrodynamic condition (step 2; 300 rpm). This system was highly suitable for the selective analysis of DA in a simulated human real sample with a recovery of 103%. It is expected that carbon–oxygen-like surface functional groups generated on the surface were responsible for the improved performance. In continuation, in 2013, Alipour et al. reported a differential pulse voltammetry (DPV)-based simultaneous detection of DA and UA using a PGE* in pH 5 PBS [49]. In this work, the author adopted 100 continuous CV cycling of PGE in a window, 1.5–2 V vs SCE in 0.1 M PBS pH 7. In further, DPV analysis of DA and UA in real samples, such as human serum, urine and pharmaceutical systems, were successfully demonstrated. Indeed, there was no effort taken to analyze the surface feature of the PGE* involved in the electrochemical analysis. In 2016, Li et al. developed a pencil-drawn homemade electrochemical sensor device using a 6B grade pencil as a working electrode (WE), counter electrode (CE) and reference electrode (RE) for DA point of care (POC) application (Figure 1A) [3]. In general, the POC refers to a portable onsite detection tool suitable for biomedical and clinical applications. The electrodes were patterned on a pre-designed Whattmann-grade filter paper by drawing the template as a layer-by-layer system until obtaining the desired resistance. As an important step, the manually prepared patterns were cured by heating at about 150°C for the electroanalytical DA sensing application (Figure 1B). Scanning electron microscope (SEM) analysis of the printed systems exhibited a porous and rough surface morphology of the cellulose fibers that might be helped for good adherence of graphitic structure on the matrix and further to an appreciable electroanalytical performance (Figure 1C,D). However, the preparation of bulk and reproducible sensor seems to be a challenging task in the above case. In 2017, Chandra et al. proposed a distinct flame itch-based surface activation method of PGE for electrochemical DPV sensing of DA [50]. The flame itching technique was carried out by exposing the PGE in flame (match stick) for a time period of 10–15 s, which resulted in a random re-alteration/carbonization of organic binders on the surface and the formation of carbon–oxygen bonds. The X-ray diffraction (XRD) analysis of the modified system reveals the formation of graphitic structures on the surface. Indeed, the CV response of the modified electrode showed a high capacitive current in the order of about ~150 µA, which is less attractive for the faradaic reaction-based electroanalytical application. Further, the deposition of unknown black particles on the PGE surface was observed during the electrode preparation, which may be an unsuitable condition for further electroanalytical application. In 2018, Mahanthesha et al. demonstrated a selective DPV-based DA detection in the presence of SE neurotransmitter using a 0.5 mm HB grade PGE in 0.2 M PBS of pH 7 [51]. There was no pretreatment procedure mentioned in this work. The CV responses showed a good electron-transfer behavior of DA with a peak-to-peak (ΔE_p_) value, ~64 mV. This system showed a concentration linearity of 5 to 40 µM and a detection limit value of 0.67 µM. The exact surface feature details for the electrochemical reaction were not explored in this work. In 2019, Fan et al. reported a surface graphenized PGE* (2B, 0.5 mm diameter, 5 mm length) for the selective DPV analysis of DA in pH 7 0.2 M PBS [52]. In a typical procedure, the PGE was coupled with platinum as a two-electrode system and was subjected to a high positive voltage, 3 V for 150 s in a 2 M NaOH solution phase condition. Based on the physicochemical characterization experiments, such as SEM and Raman spectroscopy, it has been revealed that delamination followed by graphenization of the surface occurred as an important step during the PGE surface activation. This unique feature has been found to be efficient for electroanalytical applications. In 2020, Sankaranarayanan et al. reported anodized disposable 0.5 mm HB grade PGE for the simultaneous detection of DA and UA in pH7 0.1 M PBS [39]. For the anodization procedure, an applied potential window of 2 V to 120 s was adopted in 0.1 M PBS 7. In this work, FT-IR was recorded using KBr pellet with a grinded PGE* powder. The characteristics’ peaks, 3144 cm^−1^ and 1088 cm^−1^ corresponding to –OH and –C–O, were obtained, depicting the generation of carbon–oxygen functional groups on surface. Recorded Raman spectra analysis showed characteristic D and G bands with intensity ratio, I_D_/I_G_ value for PGE = 0.589 and PGE* = 0.594, confirming the generation of disordered sp^3^ bonding due to the carbon–oxygen functional group on the surface. 

### 2.2. Polymer-Modified PGE

**Figure 2:**
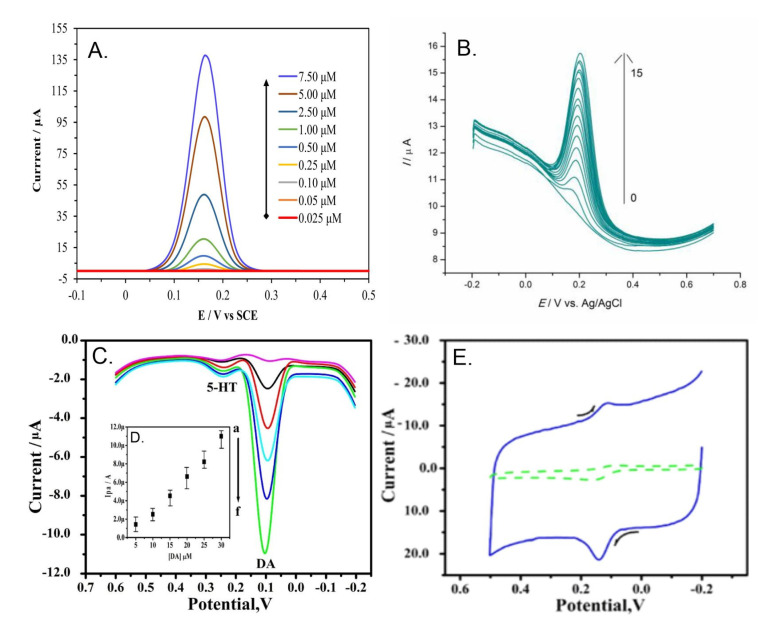
(**A**) AdsV response of PGE/p−(P3CA) in PBS (pH = 6) in the presence DA concentrations (Accumulation period = 120 s; stirring rate = 300 rpm). (**B**) DPV response recorded for PGE/4-ABSA in presence of DA concentration ranging 0.5 to 15µ.mol in PBS of pH 7. (**C**) DPV response of PGE/p-(y−PX4R) with DA concentration ranging 10−60 µM and 10 µM of SE in PBS of pH 7.4 solution, Inset (**D**) is the calibration plot of *i*_pa_ vs DA/µM. (**E**) is the CV response in presence of 10 µM DA (solid line) and in 0.1 M PBS solution (dashed line). Reprinted with permission of Elsevier publisher [40,55,58].

Owing to the unique molecular property, conductivity, easy preparation methods, highly adsorption nature, stability, flexibility and low cost, conducting and ionic polymers modified electrodes have been extensively used in biosensors and chemical sensor fabrications. Electrochemical modification of the polymeric systems on PGE surface resulted in improved electron-transfer functionality. In the literature, there are several polymer-based chemically modified electrodes (CME) reported for DA analysis. PGE has been a great choice of electrode because of its good electronic conductivity. It can be considered a good alternative to the expensive conventional GCE. Table 2 provides detailed information about the polymer-modified PGEs toward electrochemical DA analysis. In 2011, Chandra et al. reported electrochemical polymerization of fast sulfone black F (FSBF) on 0.5 mm HB PGE by potential cycling method in a window of −0.4 to 1.4 V vs. SCE in 1 mM FSBF containing 0.05 M H_2_SO_4_ as a first step and 0.1 M NaOH as a second step [53]. The FSBF is a sulphonyl group containing dye, commonly used as an indicator in colorimetric titrations. The PGE/FSBF modified electrode didn’t show any faradaic response in 0.2 M acetate buffer of pH 7 solution, whereas, in the presence of DA a well-defined quasi-reversible-like peak along with a large background current was observed. Using DPV as a sensing technique, this approach exhibited a good DA sensor response with a linear range of 0.1–0.5 µM and a detection limit value of 0.05 µM. In 2017, Koyun et al. studied a polypyrrole nanofiber (NFPPY) prepared by an overoxidized method using 0.5 mm PGE as a precursor. Electrochemical polymerization (Step-1) was carried out using 0.1 M pyrrole (PPY) as a precursor and 0.1 M LiClO_4_ + 0.1 M Na_2_CO_3_ as a supporting electrolyte [54]. Then the PPY was overoxidized by potential sweeping method at a potential window of 0 to 0.9 V vs SCE in 0.1 M NaOH solution (Step-2). The “as prepared” modified electrode was named as PGE/OO_10_NFPPY_5_, wherein the scan rates used in step-1 and 2 were placed as subscript numbers. The DA sensing experiments were performed using DPV technique. As a proof-of-concept DA containing pharmaceutical ampule was used as a real sample system. In 2017, Ozcan et al. fabricated a low-cost CME using a polypyrole-3-carboxylic acid (p-P3CA) electropolymerized over electrochemically oxidized PGE (0.5 mm) surface for the DA detection [55]. As in the first step, PGE was overoxidized at an applied potential of +1.9 V vs SCE for 45 s in 0.1 M PBS pH 8.5. As in the second step, electro-polymerization was carried out in a 20 mM P3CA containing 0.1 M LiClO_4_ and 0.1 M NaCO_3_ via electrochemical potential cycling at a potential window of 0–1.25 V vs SCE. CV responses recorded for PGE/p-P3CA in the presence of 5 µM DA showed a well-defined redox-like response at E^0^ = 0.082 V vs Ag/AgCl. DA analysis was carried out using AdSV technique in PBS (pH 6) solution (Figure 2A). In order to adsorb DA on PGE, an OCP technique was carried out with an accumulation time period of 120 s assisted with stirring (300 rpm). The modified electrode was extended to human blood and pharmaceutical, real sample analysis with an appreciable recovery percentage. 

**Scheme 4:**
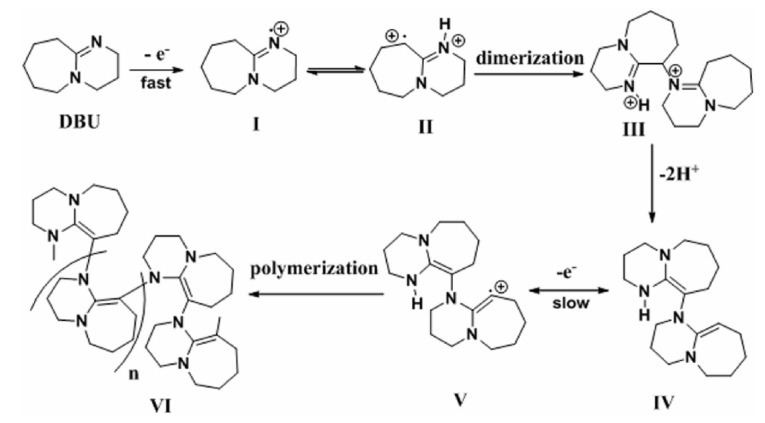
Proposed mechanism for electro polymerization of PGE/p–DBU. Reprinted with permission of Elsevier publisher [59].

In general, covalently linked modified electrodes, such as amide bond formation via carboxylic acid and amine functional group interaction, express good stability and selectivity towards desired electroanalytical applications. In 2017, Devaramani et al. reported a 4-aminobenzene sulfate (4-ABSA) modified PGE (2 mm) as an electrochemical for DA sensor [40]. In this procedure, a pristine PGE was directly exposed into a 0.1 M KCl solution containing 5 mM of 4-ABSA solution and electro-polymerized in a window of +0.5 to +1.5 V at 10 mVs^−1^ in 0.1 M KCl. During this process, the covalent linkage of an amide bond is formed between carboxylic functional group (-COOH) present on the PGE and -NH_2_ group of the monomer. The negatively charged sulphonyl group present in the 4-ABSA helped in charge repulsion with AA in the real sample for selective DA analysis (Figure 2B). PGE/ABSA electrode showed a sharp DPV signal for DA sensing with a linear range of 0.5 to 15 µM. In further, real sample analysis was demonstrated using a pharmaceutical and human urine sample. In 2018, Krishanan et al. reported poly-riboflavin (p-rB) modified PGE for simultaneous DPV based detection of SE and DA in 0.1 M PBS solution [56]. For the activation of PGE (0.5 mm), a previous literature procedure was adopted. The polymerization reaction of riboflavin was carried out using PGE by CV technique in a potential window of −1 to 1.5 V vs Ag/AgCl at *v* = 50 mVs^−1^ in 0.1 M PBS (pH 7.4). The CV response did not exhibit any redox feature. However, the PGE/p-rB electrode showed specific faradaic responses in the presence of DA and SE. A continuous increment in the peak current signals was noticed upon DA addition in DPV along with parallel baseline increments, which may be due to the co-adsorption of DA on the PGE/p-rB electrode. In 2019, Deepa et al. fabricated a poly sorbitol (p-sorb) modified PGE (0.7 mm) for electrochemical DA analysis in pH 7.4 0.2 M PBS [57]. Firstly, PGE was exposed to 0.1 M NaOH solution containing 25 mM of sorbitol and subjected to electrochemical cycling in a potential window of −0.7 to 0.7 V vs. SCE at 50 mVs^−1^. SEM analysis of the modified electrode showed uniform flake-like distributions over the surface of the electrode. Further, the DPV was recorded for varying DA concentrations, showing a linear range in a window of 10–40 µM. The modified electrode was not extended to real sample analysis. In 2020, Shashikumara et al. reported an electropolymerized yellow PX4R membrane on PGE for DA analysis [58]. For the electropolymerization reaction, a continuous potential cycling was carried out in a potential window of −0.6 to +1.6 V vs SCE at a scan rate of 100 mVs^−1^ in 0.1 M NaOH solution containing 1 mM of PX4R dye. PGE/p(y-PX4R) electrode showed a redox response towards DA. A high capacitive current in the CV response for the polymeric membrane and a feeble faradaic response were observed during the DA oxidation (Figure 2E). Further, the electroanalytical performance of the PGE/y-PX4R electrode showed a well-defined DPV response for DA in the presence of SE (Figure 2C,D) and further validated by real sample analysis. In 2021, Rejithamol et al. studied 2,3,4,6,7,8,9,10-octahydropyrimido [1,2-a] azepine (DBU) electropolymerization on PGE and its simultaneous voltammetric analyses of DA, SE and Tryptophan (typ) [59]. The electropolymerization reaction was conducted in 1 M H_2_SO_4_ medium containing 2 mM of DBU in a potential window of −0.5 to +1.5 V vs Ag/AgCl at *v* = 100 mVs^−1^. The polymerization was achieved by the cation radical generation mechanism (Scheme 4). The polymer film was physiochemically characterized by FT-IR and SEM analysis. DPV responses of PGE/p-DBU modified electrode showed a sensitive DA analysis with a linear range of 1–20 µM and a detection limit of 50 nM in 0.1 M PBS. Further, the PGE/p-DBU was extended to DA real sample analysis of the human blood serum samples. 

### 2.3. Metal Oxides and Nanocomposites Modified PGE

Metal-oxide modified PGEs are well known for their high electrochemical performance, stability, durability and good electron transfer kinetics. In this section, we have collected and presented PGE modified with metal oxides, nanoparticles and nanocomposite-based materials for DA electrochemical sensing as in Table 3. Abbreviations used in this review are tabulated at the bottom of the main text. In 2015, Imran et al. synthesized graphene oxide (GO) by electrically exfoliating 0.7 mm PGE and prepared a GCE/ErGO@AuNP CME (ErGO = Electrochemical reduced graphene oxide and AuNP = gold nanoparticle) for a DPV based simultaneous determination of DA, AA and UA in PBS (pH 7.4) [12]. First, electrical exfoliation was carried out by applying a DC potential of +7 V between two PGEs in three different solutions: HCl (acidic), PBS (neutral) and NaOH (basic). In order to confirm the ErGO formation, the synthesized ErGO was subjected to physiochemical characterizations, such as SEM, Raman spectroscopy and XRD. The results revealed that the ErGO prepared in PBS solution showed better quality than other mediums. The I_D_/I_G_ ratio values obtained from Raman analysis was correlated well with the SEM and XRD results. Further, from the synthesized ErGO powder, a 1.5 mg of ErGO was taken and dispersed in 1 mL of dimethyl formamide and then drop casted on a fresh GCE surface. For the in-situ formation of AuNPs on GCE/ErGO, an electroreduction of HAuCl_4_.nH_2_O was carried out by potential cycling in the window of −0.6 to 0 V vs Ag/AgCl for 20 cycles. After completing the processes, GCE/ErGO@AuNPs modified electrode was utilized for the simultaneous electrochemical detection of DA, UA and AA in pH 7 PBS solution. CV response of the modified electrode showed an efficient irreversible oxidation response towards DA and DPV sensing response of DA. Real sample analysis was demonstrated using different juice (for AA) and pharmaceutical samples. 

**Figure 3:**
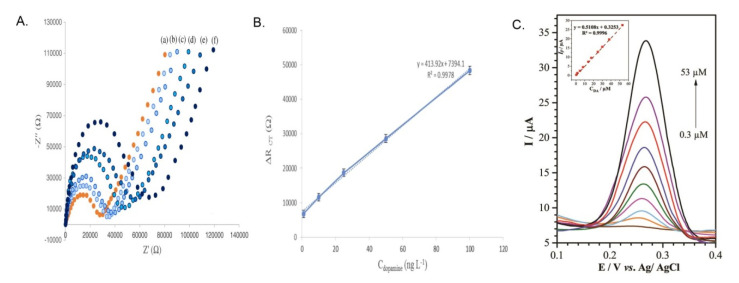
(**A**) Electrochemical impedance spectra (EIS) of PGE/GNS/DA modified electrode in [K_3_Fe(CN)_6_]/[K_4_Fe(CN)_6_] solution where (**a**) is before incubation and (**b**−**f**) are increasing concentrations of DA from 6−650 pM and (**B**) its respective calibration graph. (**C**) DPV response of the PGE/Cu/CuxONPs modified electrode in presence of DA concentration ranging 0.3−0.53 µM. Reprinted with permission of Elsevier publisher [42,60].

**Figure 4:**
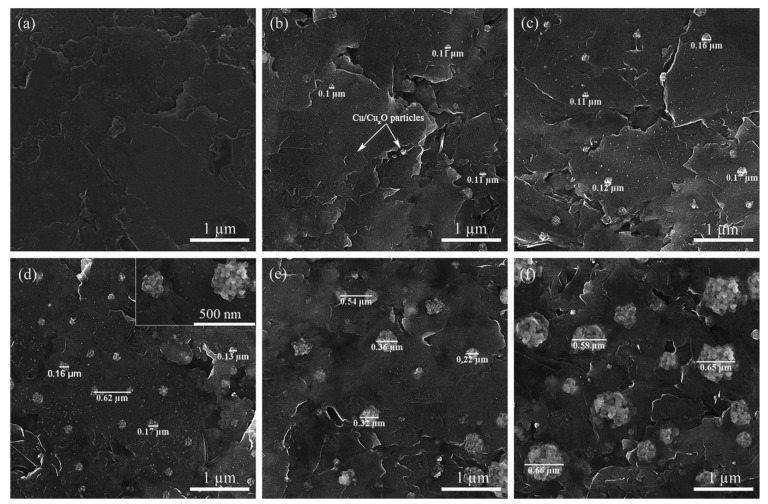
FESEM images; (**a**) a bare PGE, (**b**) a PGE modified by Cu/Cu_x_O nanoparticles electrodeposited at 50 s, (**c**)100 s, (**d**) 150 s, (**e**) 200 s and (**f**) 300 s. Reprinted with permission of Elsevier publisher [42].

**Figure 5:**
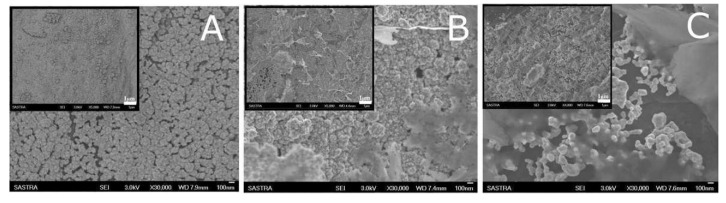
(**A**) Scanning electron microscopic images of electrodeposited copper on PGE, electrodeposited copper after silver modification using galvanic replacement (**B**) before and (**C**) after potential cycling. Inset main images before magnification. Reprinted with permission of Elsevier publisher [61].

In 2017, Talemi et al. reported a highly sensitive electrochemical impedance spectroscopy (EIS) based DA detection in K_3_Fe(CN)_6_ and NaNO_3_ (1:1) solution mixture [60]. In this work, PGE was successively modified with synthesized gold nanostars (GNS), followed by immobilizing a DA aptamer on the GNS modified surface and incubated for 40 min, resulting in a well-aligned thiol-linked self-assembled monolayer. Further, EIS experiments of PGE/GNS/DA modified electrode showed a semicircle response in the presence of K_3_Fe(CN)_6_ and NaNO_3_ (1:1) solution mixture, wherein an increase in the electron transfer resistance values was noticed during the continuous additions of DA (Figure 3A,B). Obtained EIS data were fitted into a Randles circuit model, and ΔR_CT_ values were derived from the curve-fitting approach. The proposed methodology was validated by human urine and serum real sample analysis. In 2020, Bahrami et al. reported a PGE (3 mm, 3 H) modified Cu/CuOxNPs (CuOxNPs = Copper oxide nanoparticles) for a DPV of DA analysis in pH 5.8 solution [42]. As a first step, on a fresh PGE, CuNPs were electrodeposited by the potential cycling method in a chloride-based bath containing 1 mM CuCl_2_ in 0.1 M KCl solution. Figure 4a,b shows a comparative field electron scanning electron microscope (FESEM) images of PGE and CuNPs modified PGE surfaces. It is interesting to note that the particle size of the CuNPs increased against the increase in the deposition time. The nanoparticles were found to be agglomerated on the PGE surface as shown in Figure 4c,d. Electrodeposition time of 150 s was chosen as an optimum condition. The atomic vacancy sites and cationic defects present in metal oxides are referred to be responsible factors for the facile electrochemical activity of the system. The DPV responses of the modified electrode showed a good increment over DA additions with a linear range of 0.3 to 53 µM (Figure 3C). However, no real sample analysis was performed. In 2020, Hyder et al. synthesized a Cu-Ag bimetallic nanostructure- chemically modified PGE surface (0.7 mm HB) for amperometric sensing of DA in 0.1 M PBS [61]. The electrodeposition of Cu was carried out on PGE using 15 mM of copper acetate containing 0.1 M sodium sulfate solution at an applied potential of E_app_ = −0.7 V vs Ag/AgCl using chronoamperometry (CA) technique for a time period (t = 120 s). As the second step, the PGE/Cu electrode was exposed into an AgNO_3_ (1 mM) solution, wherein the galvanic replacement reaction of copper by silver ions was achieved at OCP. The “as prepared” PGE/Cu-Ag bimetallic modified electrode was characterized by both physicochemical (SEM and XRD) and electrochemical techniques. As shown in SEM images (Figure 5A–C), the Cu-Ag bimetallic granular nanostructured (size = 20 ± 5 nm) aggregation was found to be uniform throughout the PGE surface. The modified electrode showed a good electrocatalytic performance towards DA. The amperometry i-t responses showed a step-like increase of current signals at an applied potential, E_app_ = +0.27 V vs Ag/AgCl in 0.1 M PBS solution with a linear range of 0.1–200 µM. The modified electrode was validated by real sample analysis with human urine and pharmaceutical samples. Owing to the flexible bandgap, large surface area and multiple layer structures, MXene has gained the scientific community’s attention, recently. MXenes exhibit high electrochemical performance for the detection of various analytes. In 2021, Amara et al. prepared a perlyne diimide (PDI)-MXene material-based electrocatalyst self-assembled PGE (2 mm) for DA electrochemical oxidation at a lower potential of −0.135 V vs Ag/AgCl in pH 7.4 PBS [43]. The PGE/PDI-MXene electrode exhibited a satisfactory catalytic peak current in the DPV response. The system was found to be ultrasensitive and selective for DA, even in the presence of various interfering bioanalytes. Further**,** in the same year, the group reported a copper oxide integrated PDI that has been self-assembled on a PGE for electroanalytical application [41]. Initially, the (CuONPs = Copper oxide nanomaterials) were synthesized by hydrothermal method using copper chloride dihydrate as the precursor. Further, the electrochemically pretreated PGE was immersed in PDI for 30 min, which enabled the self-assembly of PDI over PGE. As a late step, PGE/PDI was immersed again in a 20µL (3 mg/mL) of copper oxide solution for 30 min, which completed the modification of the PDI-CuONps on PGE. The modified electrodes were characterized using various physicochemical techniques. The surface morphology analysis obtained by atomic force microscopic (AFM) technique showed a thickness of 0.42 µm for PGE/PDI and 0.67 µm for PGE/PDI-CuONPs modified electrodes. The remarkable morphology difference is evident in the immobilization of CuONPs on the PGE/PDI. The PGE/PDI-CuONPs modified electrode was subjected to electrocatalytic oxidation of DA by CV and amp i-t (E_app_ = 0.57 V vs Ag/AgCl) in PBS solution, wherein a step-like increment in the current signal upon DA addition was observed in a range from 5 to 500 µM. The modified electrode was validated by human serum and pharmaceutical, real sample analyses with a recovery of ~100%. In the following year, Baig et al. reported the fabrication of graphene nanosheet (Gr_nano_) sandwiched platinum nanoparticles (PtNP) modified PGE [62]. The proposed CME was achieved by a three-step layer-by-layer sandwich preparation method. As step-1, the PGE was dipped into a GO dispersion (4 mg in 1 mL DD water) and sonicated for 30 min. A PGE-deposited GO electrode was subjected to CV in the window of −1.4 V to 0.3 V vs Ag/AgCl at *v* = 0.02 mVs^−1^ for one cycle. After the first layer of graphene deposition, as step-2, the PGE/GO was exposed into a 0.1 mM (NH_4_)_2_ PtCl_4_ solution, and CV was recorded in the window of −0.05 V and 0.3 V vs Ag/AgCl at *v* = 0.01 mVs^−1^ (for one cycle). During the course of the electrochemical reaction, the Pt^4+^ is reduced to Pt^o^-nanoparticles on the PGE/Gr_nano_ surface. This experiment was repeated to prepare multi-layered sandwich structures. The CME was used for DA sensing using the square wave voltammetry (SWV) technique. The proposed methodology was highly sensitive compared to several other PGE-based modified electrodes. Based on the above reports, it is evident that incorporating metal oxides and metal nanoparticles alleviated the sensitive and selective detection of DA in a physiological medium. All the reported works exhibited a well-defined, stable redox response relating to a facile electron transfer behavior for the electrocatalytic oxidation of DA. However, the major limitation of using Cu- and Au-based electrocatalysts are their competency towards H_2_O_2_ and O_2_ reduction reactions, which may hamper the analysis of DA.

**Table 1:** Tabular column listing pre-treated pencil graphite electrode.

S. No	Electrode	Pre-Treatment Conditions	Analytes	Tech, pH	Sensitivity/µA µM^−1^	Linear Range/µM	Ref
1.	PGE *	−0.6 to 0.9 V (CV) in 0.1 M PBS	DA, SE &UA	CV, PBS 7	0.004	100–700	[47]
2.	PGE *	−0.3 to 2.0 V (CV) in 0.1 M H_3_PO_4_	DA	AdSV, PBS 4	17.186	0.5–5	[48]
3.	PGE *	+1.5 to 2.0 V (CV) in 0.1 M PBS	DA, UA	DPV, pH 5	0.225	0.15–15	[49]
4.	Pencil drawn electrode	Paper based analytical device	DA	DPV, pH 7.4	6.91	0.1–700	[3]
5.	PGE/Flame itched	Exposing flame over PGE surface	DA	DPV, PBS 0.2 M	1.8	2500–10000	[50]
6.	PGE	No pretreatment procedure	DA	DPV, pH 7.0	0.70	15–40	[51]
7.	PGE/Graphene	3 V anodic voltage for 150 s in NaOH	DA	DPV, pH 7	20.8	0.15–45	[52]
8.	PGE *	E_app_ = 2 V for 120 s in PBS 7	DA	DPV, pH 7	34.32	1–80	[39]

PGE = pencil graphite electrode; (*) = pre-treatment; DA = dopamine; UA = uric acid; SE = serotonin; DPV = differential pulse voltammetry; AdSV = adsorptive stripping differential pulse voltammetry.

**Table 2:** Tabular column listing PGE-modified polymer electrodes.

	Electrodes	Analytes	Tech, pH	Sensitivity/µA µM^−1^	Linear Range/µM	Ref
1.	PGE/p-FSBF	DA, UA	CV, pH 7	-	0.1–0.5	[53]
2.	PGE/OO_10_NFPPY_5_	DA	DPV, pH 4	0.157	1.0–1000	[54]
3.	PGE/p-(P3CA)	DA	AdsV, pH 6	17.23	0.025–7.5	[55]
4.	PGE/4-ABSA	DA	DPV, pH 7	10.786	0.5–10	[40]
5.	PGE/p-rB	DA & SE	DPV, pH 7.4	0.919	1–320	[63]
6.	PGE/p-Sorb	DA	CV, DPV pH 7.4	0.116	10–40	[57]
7.	PGE/p(y-PX4R)	DA	DPV, pH 7.4	-	10–50	[58]
8.	PGE/p-DBU	DA, SE, Typ	DPV, PBS	20.8	1–20	[59]

p-FSBF = poly (fast sulfone black F); p-sorb = poly-sorbitol; Nf- Nanofibril; PPY- polypyrrole; P-rB-polyriboflavin; P3CA- pyrrole-3-carboxylic acid; 4-ABSA- 4-Amino benzenesulfonate; Typ-tryptophan; SE-Serotonin; p(y-PX4R) = poly (yellow PX4R); p-DBU = poly 1,8-diazabicyclo [5.4.0] undec-7ene.

**Table 3:** Tabular column listing metal and composite based modified PGE electrode.

	Electrode	Analytes	Tech, pH	Sensitivity/µA µM^−1^	Linear Range/µM	Reference
1.	GCE/ErGO-AuNPs	AA, DA & UA	DPV, pH 7.4	0.0412	0.01–3000	[12]
2.	PGE/GNS/DA	DA	EIS, (1:1) ratio of K_3_Fe(CN)_6_ and NaNO_3_	413.9 ohms	6–6.5 × 10^−6^	[60]
3.	Cu-Ag bimetallic	DA	CA, PBS 7.4	1.56	0.1–200	[61]
4.	PGE/Cu/Cu_x_ONPs	DA	DPV, pH 5.8	0.51	0.3–53	[42]
5.	PGE/PDI-CuO	DA	DPV, pH 7	4	5–50	[41]
6.	PGE/PDI/MXene	DA	DPV, pH 7	38	100–1000	[43]
7.	PGE/Gr_nano_/PtNPs/Gr_nano_	DA	DPV, pH 7	210	0.06–20	[62]

AuNPs = gold nanoparticles; PDI-perylene diamide; Gr-graphene; ErGo-electrochemically reduced graphene oxide; GNS-gold nanoclusters.

## 3. Summary and Future Perspectives

This review article has covered different PGE-based electrochemical detection methods for the DA. From the literature, we could infer the reliability of the PGE* for the sensitive and selective detection of DA. The cost-effective surface activations-based PGE* systems had shown a clear DPV signal and good selectivity with a significant peak-to-peak potential difference in the simultaneous detection of DA and other electroactive analytes. At the same time, very few reports have provided the physicochemical aspects and surface characteristics of pre-treated PGE. Some in-situ electrochemical studies, based on Raman, FT-IR and scanning electrochemical microscopy (SECM) approaches, are highly needed to access the nature of surface functionalization and electron-transfer activities of the PGE*. Further, edge plane, basal plane formation mechanism on PGE and its surface insights relating the electrocatalytic activity are important research topics future direction of PGE. In the case of electropolymerized organic monomers, a marked increment in the PGE* conductivity and electroactive surface area was noticed. For the case of metal and nanocomposite-modified PGE, even though the modified electrodes were efficient for DA electroanalysis, associated issues such as dissolved oxygen interference, pH sensitivity and nanoparticle toxicity are of great concern. Alternately, environmental begin molecular system modified PGE is an eco-friendly route for emerging electroanalytical applications. PGE can be extended for bulk and low-cost synthesis of GO suitable for energy materials, electrocatalytic oxidation/reduction, functionalized carbon nanomaterial developments, etc. Furthermore, selective and simultaneous DA analysis in the presence of other structural isomers, such as norepinephrine, epinephrine and catechol, has to be explored. PGE-based sensor devices integrating with smartphones, miniature-cell compartments, soft computation techniques, machine learning and deep learning methods will be hot research topics for DA-based point of care application systems. 

## Abbreviations

DBU	2,3,4,6,7,8,9,10-octahydropyrimido [1,2-a] azepine
4-ABSA	4-Aminobenzene sulfate
AdsV	Adsorptive stripping voltammetry
AA	Ascorbic acid
AFM	Atomic force microscopy
CME	Chemically modified electrode
CA	Chronoamperometry
CE	Counter electrode
CV	Cyclic voltammetry
DPV	Differential pulse voltammetry
DA	Dopamine
ErGO	Electrochemically reduced graphene oxide
FSBF	Fast sulphonyl black F
FESEM	Field emission electron microscopy
FT-IR	Fourier transform infra-red
GCMS	Gas-chromatography-Mass-spectrometry
GCE	Glassy Carbon electrode
GO	Graphene oxide
GNS	Gold nano stars
HPLC	High pressure liquid chromatography
NP	Nanoparticles
OCP	Open circuit potential
PGE	Pencil graphite electrode
PDI	Perylene diamide
PBS	Phosphate buffer solution
POC	Point of care application
P3CA	Polypyrole-3-carboxylic acid
NFPPY	Polypyyrole nanofibril
SECM	Scanning electrochemical microscopy
SEM	Scanning electron microscope
SE	Seritonin
SCE	Standard calomel electrode
Sorb	Sorbital
rB	Riboflavin
Typ	Tryptophan
UA	Uric acid
WE	Working electrode
RE	Reference electrode
XRD	X-ray diffraction
y-p4XR	Yellow-PX4R
